# Relationships Between Resting Energy Expenditure and Transcranial Doppler Measurements in Patients With and Without Brain Death

**DOI:** 10.7759/cureus.32093

**Published:** 2022-12-01

**Authors:** Ellada Kiourtzieva, Vasilios Grosomanidis, Ekaterini Amaniti, Dimitrios Matamis, Chryssa Pourzitaki

**Affiliations:** 1 Department of Anesthesiology and Intensive Care, Aristotle University of Thessaloniki, Thessaloniki, GRC; 2 Intensive Care Unit, Papageorgiou General Hospital, Thessaloniki, GRC; 3 Faculty of Medicine, School of Health Sciences, AHEPA University Hospital, Thessaloniki, GRC; 4 Department of Clinical Pharmacology, School of Medicine, Faculty of Health Sciences, Aristotle University of Thessaloniki, Thessaloniki, GRC

**Keywords:** transcranial doppler sonography, basal metabolic rate, resting energy expenditure, brain injury, brain death

## Abstract

Introduction

Brain metabolism deteriorates during brain death, suggesting that cerebral metabolic measurements could serve as a prognostic factor. The application of transcranial Doppler can be useful in evaluating patients evolving to brain death. Resting energy expenditure is lower than expected in patients with brain death, and this is caused by the decrease in cerebral blood flow and consequently lower oxygen supply. The primary aim of this retrospective study is to investigate the early metabolic changes in patients with clinical criteria of brain death and examine if these changes are related to a gradual decrease in blood flow velocities in the middle cerebral artery.

Methods

All consecutive patients from 1st June 2018 to 30th April 2022, admitted to the ICU with brain injury and a GCS ≤ 8, were included retrospectively in the study. Patients were allocated into two groups: Group A, patients without clinical signs of brain death (n = 32), and Group B, patients with brain death (n = 34). In each group, three sets of metabolic measurements were performed concomitantly with cerebral blood flow velocities using transcranial Doppler (a) upon admission to the ICU, (b) once hemodynamic stabilization was obtained, and (c) 48 hours after their hemodynamic stabilization or when brain death was confirmed by clinical criteria. Resting energy expenditure (REE) measurements were performed using a metabolic computer. Cerebral blood flow velocities were measured after a period of 30 min using a 2-MHZ 2D ultrasound probe.

Results

Brain-dead patients had a significant decrease in their metabolic parameters as the cerebral blood flow velocities recorded with the transcranial Doppler deteriorated, (REE Group A = 1667.65 ± 597 vs Group B = 1376.12 ± 615, p = 0.05 and REE predicted Group A = 113.19 ± 44.9 vs Group B = 93.29 ± 41.5, p = 0.066 for measurement 1; REE Group A = 1844 ± 530.9 vs Group B = 1219.97 ± 489, p < 0.001 and REE predicted Group A = 124.38 ± 39 vs Group B = 81.35 ± 30.4, p < 0.001 for measurement 2; REE Group A = 1750.97 ± 414, p < 0.001 and REE predicted Group A = 116.38 ± 19.2 vs Group B = 56.09 ± 19.6, p < 0.001 for measurement 3). Multiple stepwise regression analysis revealed a strong relationship between age, the worsening of the blood flow velocities pattern, and the decrease in REE (multiple R = 0.264, F = 5.55, p = 0.009). Furthermore, a statistically significant correlation was found between temperature and REE (correlation coefficient = 0.500, 0.674, 0.784 for measurements 1, 2, and 3, respectively, and p < 0.001 for all measures).

Conclusions

In brain-dead patients, the gradual decrease in cerebral blood flow leads to a decrease in REE as well as thermogenetic control. These changes can be detected early after the patient's admission to the ICU.

## Introduction

Currently, both clinical and laboratory criteria are used to determine whether or not a patient can be characterized as brain dead. The clinical criteria of brain death (BD) are based on the absence of brain stem reflexes and the presence of a positive apnea test. Aside from the clinical requirements of BD, some institutions consider an isoelectric electroencephalogram as a supplementary examination [1]. In difficult cases, brain angiography is essential. The clinical criteria are regarded as insufficient in patients that are hypothermic or have been sedated with large doses of anesthetic drugs. In these cases in order to appropriately diagnose BD, it is crucial to verify the absence of neuronal activity and cerebral perfusion [2-4]. The first can be documented by examining brain stem auditory evoked responses and the second using various techniques, including magnetic resonance imaging (MRI) or computed tomography (CT) angiography as well as transcranial Doppler sonography (TCD) [5-8]. TCD is a simple, bedside, non-invasive and reliable technique to estimate the absence of cerebral blood flow velocities. The increase in intracranial pressure induces distinct changes in the TCD pattern of blood flow velocities. First, the diastolic velocities are decreased, and then reverse flow patterns or systolic peak spikes are observed. In the end, as the intracranial pressure equalizes the systolic blood pressure, an absence of the intracranial blood flow signal is apparent [7,8].

The metabolic rate of an awake patient while, at rest, without physical or psychological stress is referred to as the basal metabolic rate (BMR). When the postabsorptive state is included, this represents the resting energy expenditure (REE). The REE is influenced by a variety of factors, which may alter the patient's metabolic requirements. Clinical trials investigating the changes of REE in brain-dead patients are controversial. Several studies have found a reduction in REE in brain-dead patients, probably due to hypothermia, even though brain damage causes an increase in REE when compared to BMR [9,10].

Moreover, we previously reported that REE is lower than BMR in BD patients as a consequence of the decreased cerebral blood flow and thus lower cerebral metabolism [11]. In the present study, we hypothesized that the combination of REE and TCD can be useful as an indicator of brain death in patients with brain injury. Our aim was to investigate the relationship between REE and TCD blood flow velocities in patients with brain injury and to find if the changes in REE and TCD patterns could be related to the final outcome.

## Materials and methods

Study design

This is a retrospective observational cohort study, including adult patients suffering from brain injury. The study protocol was approved by the Bioethics Committee of the School of Medicine, Faculty of Health Sciences, Aristotle University of Thessaloniki, and the study was conducted according to the Declaration of Helsinki. The trial was submitted with the acronym “SERENADE” (Resting Energy Needs in Brain-Dead Patients [reSting EneRgy nEeds iN brAin-DEad Patients]) for inclusion in the ClinicalTrials.gov PRS Registry (NCT) and has been registered and allocated the ID: NCT05070182.

Patients

From the 1st of June 2018 to the 30th of April 2022, all the patients from Papageorgiou General Hospital of Thessaloniki suffering from brain damage were enrolled retrospectively in the study. The inclusion criteria were age from 18 to 90 years old, with a GCS ≤ 8 before intubation. The additional inclusion criteria were clinical signs and CT findings of intracranial damage after (i) a traumatic brain injury, (ii) a cardiac arrest successfully resuscitated, and (iii) intracranial hemorrhage. The exclusion criteria included past history of central nervous system (CNS) disease (Alzheimer's disease, multiple sclerosis, brain tumor, and aneurysm) in the last three years and previous history of cachexia due to cancer. In all patients, a brain CT scan was already performed to assess the brain injury.

Study protocol

All patients included in the study were intubated (either at the emergency room or at the accident scene) and mechanically ventilated before entering the ICU. The patients were monitored for their clinical condition (GCS before sedation and intubation, pupillary size, and response to light, according to the anesthesiologist that had intubated them). According to our department's protocol, there was a standard approach for patients with brain pathology admitted to the ICU. This protocol included a baseline metabolic profile performed concomitantly with the TCD blood flow velocity measurements in the middle cerebral artery upon their admission to the ICU.

**Figure 1:**
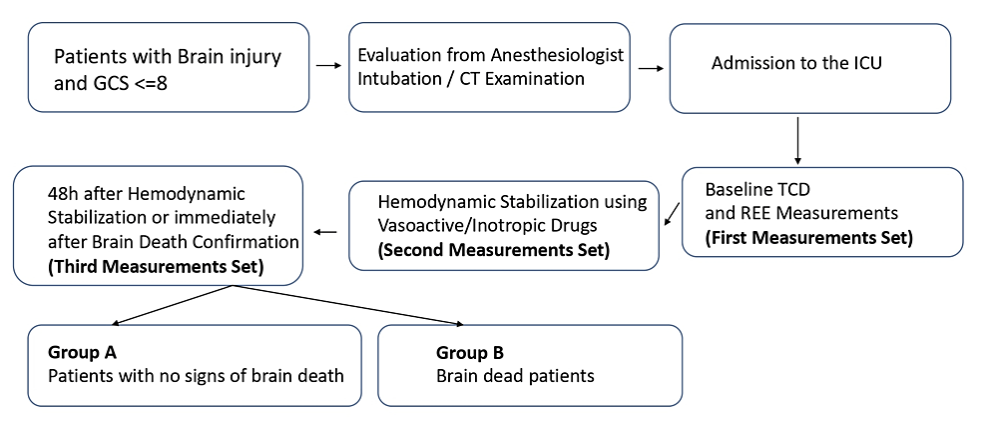
Study protocol GCS: Glasgow scale; CT: Computed tomography; ICU: Intensive care unit; TCD: Transcranial Doppler; REE: Resting energy expenditure.

According to their clinical outcome, after 30 days upon admission, the patients were divided into two groups: Group A, which included patients with intracranial pathology without BD semiology, and Group B, which included patients with BD semiology. Group A comprised the control group, and group B comprised the study group. In both groups, once hemodynamic stabilization was obtained (using vasoactive and inotropic drug infusion), the patients had a second set of measurements. The third set of measurements was conducted 48 hours after their hemodynamic stabilization or when BD was confirmed based on clinical and TCD criteria (Figure 1) [12-15].

Monitoring

In all study participants, the heart rate (HR), respiratory rate (RR), and oxygen saturation (SpO_2_) were measured along with the standard monitoring applied to critically ill patients.

Transcranial Doppler

TCD was performed at the same time with REE measurements using a low-frequency ultrasound 2-MHZ 2D ultrasound probe (Computer Sonography Accuson 128 XP/10c, USA) over a period of 30 minutes [11,12]. MCAs were found bilaterally using two-dimensional color-flow mapping and pulse-wave signals with angle correction insonating to a depth of 40-60 mm as we have previously shown [11]. If early systolic spikes and the lack of a previous TCD signal continued for more than 24 hours, they were considered as signs of BD. These observations are common when the ICP surpasses the arterial blood pressure, causing cerebral blood flow and perfusion to cease. Patients who did not have a TCD signal on admission were not included in the study because their absence could not be attributed to a lack of an acoustic window or a lack of cerebral blood flow [11,12]. These sets of measurements were chosen for statistical analysis because they were performed after hemodynamic stabilization and before other factors like nosocomial infection could influence REE. After the third set of measurements, the patients were categorized as non-brain dead (Group A) and brain dead (Group B).

Resting energy expenditure

In all ICU patients included in the study, their REE was recorded. REE was measured by indirect calorimetry using the metabolic computer (Medical Graphics, Ultima CCM™, Minneapolis, USA) connected to the ventilator [10,11]. Before each REE measurement, the calibration of the metabolic computer pneumotachograph was performed using a three-liter syringe with an acceptable error below 9 ml/lit. The metabolic unit was calibrated for VO_2_ measurements with two calibration cylinders containing 21% and 12% O_2_. The calibration for VCO_2_ measurements was conducted with calibration gases containing 5% and 0% CO_2_, respectively [10-13]. The metabolic computer continuously recorded the breath-to-breath volume (Vt), RR, the ventilation per minute volume (MV) as well as the inhaled and exhaled gases (O_2_ and CO_2_). Using those measures, it has the ability to calculate the values ​​of VO_2_, VCO_2_, and also the values ​​of REE and respiratory quotient (RQ) [1,3,8,11]. This method calculates REE based on the measurement of oxygen consumption (VO_2_) and carbon dioxide production (VCO_2_) in the expired gases according to the Weir equation: REE = [(3.94 x VO_2_) + (1.1 x VCO_2_)] x 1,440 [15,16].

Study parameters

The parameters measured in all groups were the temperature (TEMP), the REE, the predicted REE (REE Pred), VO_2_, VCO_2_, RQ, inspired oxygen concentration (FiO_2_), RR, and SpO_2_. Moreover, the blood flow velocities in the middle cerebral artery were measured in all patients for both sides (right R and left L) as peak systolic velocity (PSV), end-diastolic velocity (EDV), and mean diastolic flow velocity (MDV). Additionally, the pulsatility index (PI) provided by the software ECHO machine was recorded.

Study outcomes

The REE and REE predicted difference between the two groups, the difference in flow rates in the median cerebral artery (MCA), and the correlation of the REE changes with those in flow rates in the MCA were set as the primary outcomes of the present study. The differences between VO_2_, VCO_2_, RQ, FiO_2_ inhaled oxygen concentration, RR, HR, SpO_2_, and PI were set as the secondary outcomes. All the parameters were evaluated using three measurements with a time frame from day 0 (upon entry to ICU) to day 30.

Statistical analysis

The sample size was calculated (after pilot patients) using the G*Power 3.1.7 program. Taking REE measures as the primary parameter, it was estimated that a total sample of 62 patients was needed for obtaining an α = 0.05 and power (1-β err prob = 0.8), while the effect size "f" was set at 0.3. Prior to any analysis, the data from all parameters at each measurement time was checked for the regularity of the distribution with the Kolmogorov-Smirnov distribution control test. For the analysis of the demographic data, the student’s t-test method was used for the comparison of quantitative demographics of the study groups and the chi-square control for the corresponding comparison of the qualitative characteristics (e.g., gender). One-way analysis of variance (ANOVA), simple regression with the Spearman test for nonparametric data, and multiple stepwise regression analysis were also used for the serial measurements of temperature, REE, and TCD measurements in all patients. For statistical analysis, the SPSS v27.0 software (IBM Corp., Armonk, NY) for windows was used. The significance level was set at 0.05 or 5%.

## Results

In total, 66 patients were included in the study protocol. The Kolmogorov-Smirnov test indicated a normal distribution pattern for all variables. Table 1 summarizes the patients’ characteristics and their mean values (± standard deviation) for age and sex, brain pathology (traumatic brain injury, cardiac arrest successfully resuscitated, or intracranial hemorrhage), days of stay in mechanical ventilation, and total length of stay (LOS) in the ICU. Statistical analysis revealed that there was no significant difference between groups either for sex, age, or brain pathology parameters. However, there was a statistically significant difference between groups in mechanical ventilation days (p < 0.001) as well as in LOS (p < 0.001).

**Table 1:** Patient characteristics

			Group Α (n = 32)	Group Β (n = 34)	Significance
Age (years)			54.8 ± 21.2	56.7 ± 14.3	p = 0.67
Sex (Male/Female)			17/15	19/15	p = 0.509
Brain pathology					
Traumatic brain injury			9 (28%)	12 (35.3%)	p = 0.526
Cardiac arrest successfully resuscitated			12 (37.5%)	8 (23.5%)	p = 0.203
Intracranial hemorrhage			11 (34.5%)	14 (41.1%)	p = 0.584
Time in mechanical ventilation (days)			12.7 ± 3.6	8.2 ± 3.6	p < 0.001
Length of stay in the intensive care unit (days)			15 ± 3.2	8.9 ± 4.4	p < 0.001

In Table 2, the results from the measurements of the flow velocities in the middle cerebral artery on both sides are shown as mean values and standard deviation. The third TCD and REE measurements were conducted between the third and fourth days after their admission to the ICU.

**Table 2:** Flow velocities in the middle cerebral artery PSV: Peak systolic velocity (right and left); EDV: End-diastolic velocity (right and left); MDV: Mean diastolic flow velocity (right and left); PI: Pulsatility index (right and left).

	Measurement	Group Α (n = 32)	Group Β (n = 34)	Significance
PSV right	1	87.2 ± 46.8	86.4 ± 49.9	p = 0.9
	2	104.8 ± 56.76	46.3 ± 10.5	p < 0.001
	3	99.7 ± 62	Flow absent	NA
PSV left	1	81.4 ± 50.5	75.4 ± 43.2	p = 0.7
	2	75.4 ± 43.2	58.9 ± 24.6	p = 0.45
	3	93.5 ± 56.1	Flow absent	NA
EDV right	1	34.4 ± 27.4	18.8 ± 20.9	p = 0.066
	2	38.8 ± 25.5	8.5 ± 9.7	p < 0.001
	3	37 ± 19.2	Flow absent	NA
EDV left	1	28.6 ± 18.8	18.3 ± 12.2	p = 0.021
	2	33.1 ± 25.8	11.2 ± 13.5	p = 0.15
	3	37.1 ± 25	Flow absent	NA
MDV right	1	27.6 ± 25.1	15 ± 19.6	p = 0.041
	2	33.8 ± 25.3	6 ± 9.5	p = 0.010
	3	35.2 ± 21.7	Flow absent	NA
MDV left	1	25.8 ± 16.8	14.7 ± 13.3	p = 0.008
	2	26.6 ± 22	8.6 ± 13.9	p = 0.18
	3	36 ± 24.6	Flow absent	NA
PI right	1	1.2 ± 20.4	2.7 ± 2.35	p < 0.001
	2	1.3 ± 0.4	2.4 ± 1.5	p = 0.001
	3	1.3 ± 0.3	Flow absent	NA
PI left	1	1.1 ± 0.5	2.1 ± 1.1	p < 0.001
	2	1.1 ± 0.6	2.6 ± 1.6	p = 0.001
	3	1 ± 0.4	Flow absent	NA

**Figure 2:**
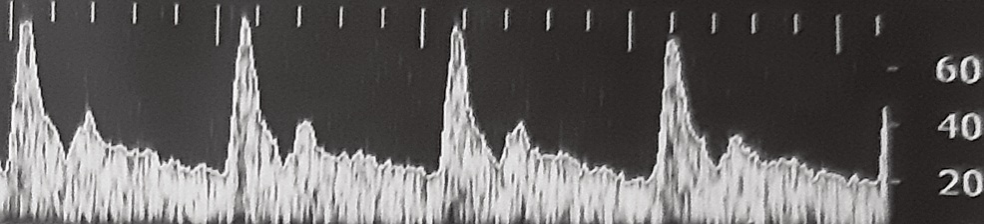
Transcranial Doppler from a Group A patient with normal findings during the first measurement

**Figure 3:**
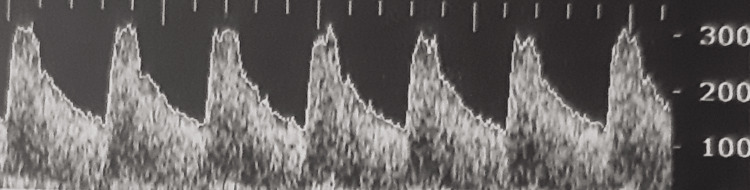
Transcranial Doppler from a Group B patient with vasospasm during the second measurement

**Figure 4:**
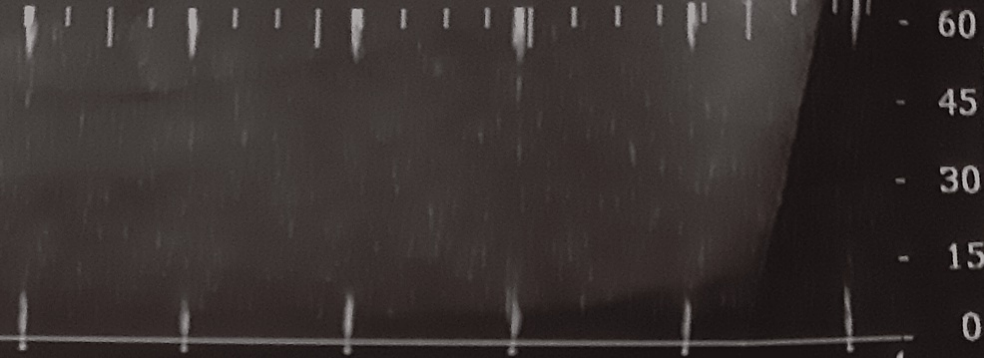
Transcranial Doppler (left side) from a Group B patient during the third measurement. The brain death was confirmed, and the reverse of flow was apparent.

**Figure 5:**
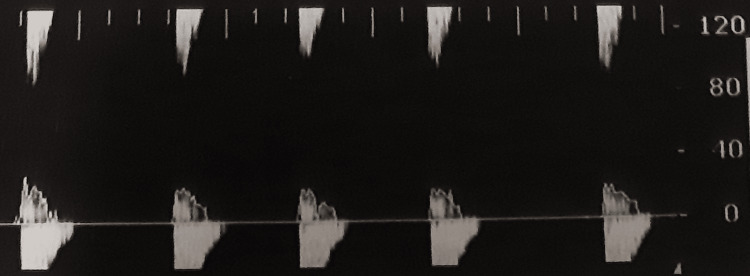
Transcranial Doppler (right side) from a Group B patient during the third measurement after brain death was confirmed and the systolic spikes were apparent.

In Group B, patients had lower peak systolic velocity (PSV) since the first measurement; however, the statistical analysis did not reveal any significance. The reduction in PSV was more apparent in the second measurement (p < 0.001). Moreover, the end-diastolic velocity (EDV) was also lower in Group B patients (p = 0.066 for the right side and p = 0.021 for the left side) in the first measurement (p = 0.021), while this difference became larger in the second measurement (p < 0.001). Patients from Group B also had lower mean diastolic flow velocity (MDV) (p = 0.008) in the first measurement, and this difference remained during the second measurement too (p = 0.01). During the third measurement, a total absence of flow was noticed in Group B, so all the flow velocities could be encountered as zero for these patients, resulting in even greater statistically significant difference for those parameters compared with Group A. Examples from TCD measurements are shown in Figures 2-5. Figure 2 shows a normal TCD from a Group A patient during the first measurement; Figure 3 reveals a TCD showing vasospasm in a patient from Group B during the second measurement. Figures 4, 5 show the TCD findings after BD reverse flow on the left side and systolic spikes on the right side, respectively.

Concerning the transcranial PI, patients from Group B had significantly higher values on both sides in the first and second measurements (p < 0.001 and p = 0.001 for right and left, respectively).

In Table 3, the results from the measurements of the temperature, REE, and REE predicted for both groups A and B are shown. Both group patients did not have differences in their temperature upon the first measurement. However, in the second and third measurements, Group A had a significantly higher temperature than Group B.

**Table 3:** Temperature, REE, and REE-predicted measurements REE: Resting energy expenditure.

	Measurement	Group Α (n = 32)	Group Β (n = 34)	Significance
Temperature	1	36.9 ± 1.1	36.6 ± 1.2	p = 0.12
	2	38.1 ± 0.9	36.4 ± 1.2	p < 0.001
	3	37.8 ± 0.9	35.8 ± 1.1	p < 0.001
REE	1	1667.65 ± 597.8	1376.12 ± 615.2	p = 0.05
	2	1844.03 ± 530.9	1219.97 ± 489.8	p < 0.001
	3	1750.97 ± 414.7	796.754 ± 319.9	p < 0.001
REE predicted	1	113.19 ± 44.9	93.29 ± 41.5	p = 0.066
	2	124.38 ± 39.8	81.35 ± 30.4	p < 0.001
	3	116.38 ± 19.2	56.09 ± 19.6	p < 0.001

The REE and REE-predicted measurements showed a marginally significant difference in the first measurement where Group A had higher REE values. Additionally, in the second and third measurements, the patients from Group B had significantly lower values of REE and REE predicted. Also, Group B patients had a significant decrease in their metabolic parameters as the cerebral blood flow velocities recorded with the transcranial Doppler deteriorated (REE Group A = 1667.65 ± 597 vs Group B = 1376.12 ± 615, p = 0.05 and REE predicted Group A = 113.19 ± 44.9 vs Group B = 93.29 ± 41.5, p = 0.066 for measurement 1; REE Group A = 1844 ± 530.9 vs Group B = 1219.97 ± 489, p < 0.001 and REE predicted Group A = 124.38 ± 39 vs Group B = 81.35 ± 30.4, p < 0.001 for measurement 2; REE Group A = 1750.97 ± 414 p < 0.001 and REE predicted Group A = 116.38 ± 19.2 vs Group B = 56.09 ± 19.6, p < 0.001 for measurement 3).

In Table 4, the results from the measurements of VO_2_, VCO_2_, and RQ are shown. Concerning the FiO_2_ inhaled oxygen concentration, RR, and SpO_2_, during the first and second measurements, there were no statistically significant differences between Groups A and B (p > 0.05). According to our results, Group B patients had statistically significant lower VO_2_ and VCO_2_ since the time of the first measurement. This difference becomes more apparent and highly significant at the time points of the second and third measurements, while Group B patients had significantly higher values at the third measurement (p < 0.001) considering the RQ. 

**Table 4:** Oxygen consumption measurements VO_2_: Oxygen consumption; VCO_2_: Carbon dioxide production; RQ: Respiratory quotient.

	Measurement	Group Α (n = 32)	Group Β (n = 34)	Significance
VO_2_	1	254.9 ± 107.9	199.4 ± 91.3	p = 0.027
	2	290.9 ± 76.9	177.1 ± 82.5	p < 0.001
	3	281.3 ± 77.4	120.8 ± 48.6	p < 0.001
VCO_2_	1	212.4 ± 69.2	171.3 ± 78.1	p = 0.027
	2	247.7 ± 58.9	160.6 ± 69.2	p < 0.001
	3	240.4 ± 47.5	119 ± 38.4	p < 0.001
RQ	1	0.92 ± 0.28	0.86 ± 0.14	p = 0.29
	2	0.89 ± 0.18	0.93 ± 0.22	p = 0.37
	3	0.89 ± 0.11	1.1 ± 0.3	p < 0.001

In Group B patients, a significant decrease was observed in REE and REE predicted as the TCD measurements deteriorated, using Spearman’s correlation analysis. Specifically, even from the first measurement (obtained upon patients’ admission to ICU), the EDV and the MDV lower values were positively correlated with lower REE predicted (correlation coefficient = 0.331, p = 0.012 and 0.328, p = 0.013, respectively). Concerning the relationship between TCD measurements, REE, age, and temperature, multiple stepwise regression analysis revealed a strong relationship between increased age, the worsening of the TCD pattern (EDV and MDV), and the decrease in REE (multiple R = 0.264, F = 5.55, p = 0.009). Also, a statistically significant correlation was found between temperature and REE (correlation coefficient = 0.500, 0.674, and 0.784 for measurements 1, 2, and 3, respectively, and p < 0.001 for all measures) and REE predicted (correlation coefficient = 0.471, 0.713, and 0.797 for measurements 1, 2, and 3, respectively, and p < 0.001 for all measures). On the contrary, no relationship was found between TCD patterns and temperature.

## Discussion

In this retrospective study, 66 patients suffering from brain damage were evaluated to determine the utility of combined early REE measurements and TCD flow patterns as predictors of BD. Considering the statistically significant difference between groups, in the days staying in mechanical ventilation, as well as in LOS, the statistically significant increased time periods in Group A patients are due to the rapid deterioration of brain-dead patients from Group B leading them to death.

In addition, Group B (brain dead) patients included in our study had significantly lower REE and REE predicted since the first measurement upon admission in the ICU. This difference was further increased in the second and third measurements where actually the patients from Group B had 50% lower values compared to Group A patients. The decrease in REE values was also strongly correlated with the EDV and MDV measurements, obtained from TCD. Our findings suggest that the decreased REE is justified both by the reduction in cerebral metabolism due to the decrease in cerebral blood flow and the hypothalamic dysregulation.

Moreover, the results of the present study are in accordance with those from previously published studies showing that brain metabolism deteriorates during BD and suggesting that cerebral metabolic measurements could serve as a prognostic factor [10]. REE increases, after a traumatic brain injury (TBI), due to endogenous catecholamine release. Therefore, lately, a number of clinical studies using sympathetic blocking agents for patients with TBI have been conducted [10]. Moreover, the decrease in REE as patients deteriorate to BD has been attributed to hypothermia, while the core temperature influences the REE values [10]. Albeit, earlier investigations showed a higher than predicted REE in brain-dead patients, suggesting that energy expenditure requirements may not be met in fasting donors awaiting organ removal [17].

In 2004, the American Academy of Neurology suggested that TCD can be useful in evaluating BD conditions (sensitivity: 91%-100% and specificity: 97%-100%), according to a level of evidence IIA [18]. The sensitivity was lower (89%) in the meta-analysis of Chang et al., which included 22 stud­ies about the efficacy of TCD ultrasonography in the diagnosis of BD, but the authors concluded that TCD is a highly reliable confirmatory test for the diagnosis of brain death [12,19].

Group A patients of our study had a statistically significant higher temperature compared to Group B as well as higher REE and REE predicted in the second and third measurements. The body temperature often rises within 24 hours of a head injury, most likely as a result of hypothalamic changes and interleukin-1 elevation [20]. The temperature difference found in our study was also confirmed by the positive correlation between REE and temperature. This finding agrees with the existing literature suggesting a 10% increase in REE per 1-degree increase in body temperature [11,21]. Brain-dead patients suffer from hypothalamic failure that leads to dysregulation of central temperature control, thus explaining the hypothermia observed in patients from Group B. This finding is in accordance with the literature that suggests hypothermia is a significant determinant of decreases in REE during brain death [22].

Although Zhou et al. in 2019 found that the PI was not correlated with BD, patients from Group B had significantly higher values of PI in our case in the TCD flow pattern scanned in both the right and left middle cerebral arteries during the two first measurements [13]. This disagreement is possibly explained by the fact that our PI measurements were conducted early on the patients (one to four days after ICU admission), while the time period ranged between the first and fifth days after ICU admission in Zhou et al.'s study [13].

Considering the indirect calorimetry measures in our study, Group B patients had statistically significant lower VO_2_ and VCO_2_ since the time of the first measurement, resulting in a highly significant difference in the next two measurements. In addition, the RQ measurements between groups did not reveal any statistical difference during the first two measurements; however, the Group B patients showed a statistically significant increase at the third measurement. Our findings suggest that REE changes could be possibly attributed to lower oxygen and carbon dioxide use from brain cells in Group B patients. These results also agree with the study of Chieregato et al. that correlated the elevated RQ with early death and more severe brain injury [23].

There are certain limitations to our study such as its retrospective study design and the small number of patients included in each group; however, these are due to the difficulties in conducting a prospective clinical trial involving brain-injured patients. Additionally, during the third measurement, many brain-dead patients from Group B had an absence of flow signal in the TCD, so no comparisons could be performed. However, the statistical difference in the TCD and REE values between the two groups is already present in the first two measurements.

Our findings are in accordance with the literature and indicate that the decrease in cerebral blood flow causes multiple hypothalamic and mesencephalic dysregulations. This also leads to a decrease in REE as well as in thermogenetic control in brain-dead patients [10]. Nevertheless, according to Ronconi et al., there are only a few published studies that confirm the value of TCD before the application of the clinical protocol in order to evaluate brain death [24-29]. In addition, the use of early TCD as a predictive tool for the diagnosis of BD has been contested by Sharma who expressed serious doubts about its superiority against the clinical examination [30].

## Conclusions

Our study showed that REE and TCD patterns could be related to the final outcome in brain-injured patients. According to our results, the decrease in cerebral blood flow is correlated with the deterioration of REE in brain-dead patients. Moreover, the findings of this study confirmed the predictive value of early TCD, combined with REE measurements, in patients suffering from brain injury during the first hours after the ICU admission and after hemodynamic stabilization.

We conclude that if the intensivists combine the TCD and REE measurements concomitantly and take the temperature of the patients into account, they could safely predict the unpropitious fact of BD. When those measurements show significantly decreased TCD flow velocities and decreased REE combined with the relevant clinical signs, the BD diagnosis is probable. In the future, more prospective, randomized, and blinded studies should be conducted in order to give evidence of TCD and REE combination in predicting BD.

## References

[1] Paolin A, Manuali A, Di Paola F (1995). Reliability in diagnosis of brain death. Intensive Care Med.

[2] Firsching R, Frowein RA, Wilhelms S, Buchholz F (1992). Brain death: practicability of evoked potentials. Neurosurg Rev.

[3] Erbengi A, Erbengi G, Cataltepe O, Topcu M, Erbas B, Aras T (1991). Brain death: determination with brain stem evoked potentials and radionuclide isotope studies. Acta Neurochir (Wien).

[4] Roosen K, Tonn JC, Burger R, Schlake HP (1992). [Diagnosis of brain death]. Zentralbl Chir.

[5] Bonetti MG, Ciritella P, Valle G, Perrone E (1995). 99mTc HM-PAO brain perfusion SPECT in brain death. Neuroradiology.

[6] Lemmon GW, Franz RW, Roy N, McCarthy MC, Peoples JB (1995). Determination of brain death with use of color duplex scanning in the intensive care unit setting. Arch Surg.

[7] Jalili M, Crade M, Davis AL (1994). Carotid blood-flow velocity changes detected by Doppler ultrasound in determination of brain death in children. A preliminary report. Clin Pediatr (Phila).

[8] Pistoia F, Johnson DW, Darby JM, Horton JA, Applegate LJ, Yonas H (1991). The role of xenon CT measurements of cerebral blood flow in the clinical determination of brain death. AJNR Am J Neuroradiol.

[9] Chiolero RL, Thorin D, Schutz Y, Jequier E (1990). [Energy metabolism and craniocerebral injury]. Ann Fr Anesth Reanim.

[10] Cankayali I, Demirağ K, Kocabaş S, Moral AR (2009). Thermogenic and metabolic response to amino acid solution in brain-dead patients. Ulus Travma Acil Cerrahi Derg.

[11] Bitzani M, Matamis D, Nalbandi V, Vakalos A, Karasakalides A, Riggos D (1999). Resting energy expenditure in brain death. Intensive Care Med.

[12] Kasapoğlu US, Haliloğlu M, Bilgili B, Cinel İ (2019). The role of transcranial Doppler ultrasonography in the diagnosis of brain death. Turk J Anaesthesiol Reanim.

[13] Zhou J, Li J, Ye T, Zeng Y (2019). Ultrasound measurements versus invasive intracranial pressure measurement method in patients with brain injury: a retrospective study. BMC Med Imaging.

[14] Greer DM, Shemie SD, Lewis A (2020). Determination of brain death/death by neurologic criteria: the world brain death project. JAMA.

[15] Lewis A, Kirschen MP (2021). Brain death/death by neurologic criteria determination. Continuum (Minneap Minn).

[16] Weir JB (1949). New methods for calculating metabolic rate with special reference to protein metabolism. J Physiol.

[17] Hergenroeder GW, Ward NH, Yu X, Opekun A, Moore AN, Kozinetz CA, Powner DJ (2013). Randomized trial to evaluate nutritional status and absorption of enteral feeding after brain death. Prog Transplant.

[18] Sloan MA, Alexandrov AV, Tegeler CH (2004). Assessment: transcranial Doppler ultrasonography: report of the Therapeutics and Technology Assessment Subcommittee of the American Academy of Neurology. Neurology.

[19] Chang JJ, Tsivgoulis G, Katsanos AH, Malkoff MD, Alexandrov AV (2016). Diagnostic accuracy of transcranial Doppler for brain death confirmation: systematic review and meta-analysis. AJNR Am J Neuroradiol.

[20] Matthews DS, Bullock RE, Matthews JN, Aynsley-Green A, Eyre JA (1995). Temperature response to severe head injury and the effect on body energy expenditure and cerebral oxygen consumption. Arch Dis Child.

[21] Sztark F, Thicoïpe M, Masson F, Lassié P, Favarel-Garrigues JF, Petit-Jean ME (1993). Metabolic status of brain-dead patients managed for organ procurement. Transplant Proc.

[22] Essien EO, Fioretti K, Scalea TM, Stein DM (2017). Physiologic features of brain death. Am Surg.

[23] Chieregato A, Marchi M, Compagnone C, Albarello V, Fainardi E, Tagliaferri F, Targa L (2005). Estimated cerebral respiratory quotient and arteriovenous differences of CO2 in the ultra early detection of global ischemia in severe head injury. Acta Neurochir Suppl.

[24] Ronconi KA, Amorim RL, Paschoal FM Jr (2021). Transcranial Doppler: a useful tool to predict brain death still not confirmed by clinical assessment. Transplant Proc.

[25] Orban JC, El-Mahjoub A, Rami L, Jambou P, Ichai C (2012). Transcranial Doppler shortens the time between clinical brain death and angiographic confirmation: a randomized trial. Transplantation.

[26] Riggs BJ, Cohen JS, Shivakumar B (2017). Doppler ultrasonography of the central retinal vessels in children with brain death. Pediatr Crit Care Med.

[27] Fages E, Tembl JI, Fortea G, López P, Lago A, Vicente JL, Vilchez JJ (2004). [Clinical usefulness of transcranial Doppler in diagnosis of brain death]. Med Clin (Barc).

[28] Lampl Y, Gilad R, Eschel Y, Boaz M, Rapoport A, Sadeh M (2002). Diagnosing brain death using the transcranial Doppler with a transorbital approach. Arch Neurol.

[29] Ojha BK, Jha DK, Kale SS, Mehta VS (2005). Trans-cranial Doppler in severe head injury: evaluation of pattern of changes in cerebral blood flow velocity and its impact on outcome. Surg Neurol.

[30] Sharma D (2011). Early TCD monitoring in brain death: what may be relevant?. Neurol Sci.

